# Pruning Wound Protection Products Induce Alterations in the Wood Mycobiome Profile of Grapevines

**DOI:** 10.3390/jof9040488

**Published:** 2023-04-19

**Authors:** Giovanni Del Frari, Marie Rønne Aggerbeck, Alex Gobbi, Chiara Ingrà, Lorenzo Volpi, Teresa Nascimento, Alessandra Ferrandino, Lars Hestbjerg Hansen, Ricardo Boavida Ferreira

**Affiliations:** 1 LEAF—Linking Landscape, Environment, Agriculture and Food—Research Center, Associated Laboratory TERRA, Instituto Superior de Agronomia, Universidade de Lisboa, Tapada da Ajuda, 1349-017 Lisboa, Portugal; 2 Department of Environmental Science, Aarhus University, 4000 Roskilde, Denmark; 3 Department of Plant and Environmental Sciences, University of Copenhagen, Thorvaldsensvej 40, 1871 Frederiksberg, Denmark; 4 Department of Agricultural, Forestry, Food Sciences (DISAFA), University of Turin, Largo P. Braccini, 2, Grugliasco, 10095 Torino, Italy

**Keywords:** *Vitis vinifera*, microbiome, grapevine trunk diseases, Cabernet Sauvignon, Syrah, *Trichoderma*, copper, fungicides, biological control

## Abstract

Fungal pathogens involved in grapevine trunk diseases (GTDs) may infect grapevines throughout their lifetime, from nursery to vineyard, via open wounds in stems, canes or roots. In vineyards, pruning wound protection products (PWPPs) offer the best means to reduce the chance of infection by GTD fungi. However, PWPPs may affect non-target microorganisms that comprise the natural endophytic mycobiome residing in treated canes, disrupting microbial homeostasis and indirectly influencing grapevine health. Using DNA metabarcoding, we characterized the endophytic mycobiome of one-year-old canes of cultivars Cabernet Sauvignon and Syrah in two vineyards in Portugal and Italy and assessed the impact of established and novel PWPPs on the fungal communities of treated canes. Our results reveal a large fungal diversity (176 taxa), and we report multiple genera never detected before in grapevine wood (e.g., *Symmetrospora* and *Akenomyces*). We found differences in mycobiome beta diversity when comparing vineyards (*p* = 0.01) but not cultivars (*p* > 0.05). When examining PWPP-treated canes, we detected cultivar- and vineyard-dependent alterations in both alpha and beta diversity. In addition, numerous fungal taxa were over- or under-represented when compared to control canes. Among them, *Epicoccum* sp., a beneficial genus with biological control potential, was negatively affected by selected PWPPs. This study demonstrates that PWPPs induce alterations in the fungal communities of grapevines, requiring an urgent evaluation of their direct and indirect effects on plants health with consideration of factors such as climatic conditions and yearly variations, in order to better advise viticulturists and policy makers.

## 1. Introduction

Pruning wound protection products (PWPPs) are increasingly applied in vineyards across the globe in an attempt to reduce infection by pathogens associated with grapevine trunk diseases (GTDs). GTDs are caused by a diverse array of fungi, the most widespread of which belong to the families *Botryosphaeriaceae*, *Diatrypaceae*, *Nectriaceae*, *Phaeomoniellaceae*, *Togniniaceae* and *Hymenochaetaceae* [1,2]. The large pathogen diversity complicates the achievement of a control strategy applicable to all GTDs. This is due to the differing tolerance to active ingredients, the lack of adequate delivery methods and the poor efficacy of known agronomical practices [3]. For this reason, the scientific community recommends an integrated pest management strategy with the aim of producing asymptomatic and non-infected rooted cuttings and preventing infections from occurring in the vineyard, combining nursery sanitation procedures with pruning wound protection. Nowadays, it is well known that infections may take place in nurseries, either during the various steps of grafting and storage or due to infected propagation material [1,4]. While adequate control measures are being investigated [5,6,7], an optimal sanitary status of rooted cuttings does not prevent infection from occurring in the vineyard. Here, grapevines (*Vitis vinifera* L.) are subjected to yearly pruning, which creates numerous points of entry for fungal propagules (i.e., pruning wounds) [8]. Therefore, scientific investigation has focused on the evaluation of strategies to protect pruning wounds by applying selected products [9,10,11,12,13,14,15].

There are three main PWPP categories: inert compounds, contact and systemic fungicides and biological control agents [16]. Inert compounds such as mastics, paints and pastes are generally regarded as an effective means of protecting pruning wounds. However, their application is time-consuming, labor-intensive and expensive overall. As an environmentally friendly approach, it may only be suitable for small vineyards and not easily implemented in large-scale environments [1]. A comprehensive list of fungicides tested in pruning wound protection can be found in the reviews by Mondello et al. (2018) and Gramaje et al. (2018). Among contact fungicides, copper-based chemicals are certainly the most popular, having been employed in vineyards for well over a century. However, recent concerns about their negative effects on the environment have led to policies, such as European regulation 2018/1981, that aim to reduce their use in vineyards [17,18]. Experimental evidence suggests that its efficacy in protecting pruning wounds is limited [9]; nevertheless, copper remains a popular choice among viticulturists. Among synthetic fungicides, some of the most effective active ingredients are fluazinam, benomyl, thiophanate methyl, pyraclostrobin and tebuconazole [3,10,16]. Their efficacy varies depending on the pathogens tested, along with the protection time bracket that they offer. While not an environmentally friendly choice, fungicides offer the advantage of some degree of automation during the application process. Among natural fungicides, chitosan has demonstrated potential for pruning wound protection against some GTD fungi [12]. Numerous biological control agents (BCAs) have been evaluated as potential control strategies against GTDs [10,19,20,21,22]. In pruning wound protection, the most promising belong to the genus *Trichoderma* [5,23,24,25,26]. BCAs offer the advantage of being an environmentally friendly alternative to fungicides, since, to date, they have not been proven harmful to grapevines or the environment, they have competitive costs and their distribution can be easily mechanized [1,16]. Nevertheless, BCA-treated pruning wounds may remain susceptible to pathogen infection during multiple days after treatment [1], and there is no single BCA capable of antagonizing all GTD-associated fungi.

Interest in the plant-associated microbiome has been gaining momentum, as molecular tools such as next-generation sequencing (NGS) allow the assessment of microbial dynamics in response to biotic and abiotic factors and anthropic intervention. Plant–microbiome interactions are especially important, as numerous microorganisms have been proven critical for the well-being of plants, providing access to nutrients and helping the plant to deal with stressors [27,28,29]. In the literature, there are numerous examples of plant protection products being harmful to the environment, inducing phytotoxic effects and negatively effects on non-target organisms [30]. Within this context, recent research has revealed that fungicide application causes alterations in the microbiome profile of maize (*Zea mays*), soybean (*Glycine max*) and grapevine phyllosphere [31,32]. In grapevine, Del Frari et al. (2019b) showed that plant protection products commonly employed in vineyards against powdery and downy mildew agents (*Erysiphe necator* and *Plasmopara viticola*, respectively), when applied to vine leaves, induce alterations in the microbial composition of wood fungal endophytes [30]. In their article, the authors suggest that the use of fungicides, dating back ~150 years, may have caused a lasting imbalance in the vines’ endophytic microbiome, which contributed to the recent success of GTD pathogens [30,33].

Increasing evidence suggests that microbial homeostasis plays a major role in plant health, highlighting the urgency of an accurate analysis of possible non-target effects of some established and novel PWPPs at the community level. To this end, in this first-of-its-kind study, we selected two grapevine cultivars (Cabernet Sauvignon and Syrah) and two vineyards in Portugal and Italy and used NGS (i) to characterize the wood mycobiome of one-year-old canes and (ii) to evaluate how the endophytic fungal community responds to the application of four PWPPs. We selected Cuprocol^®^, a copper-oxychloride-based contact fungicide; Tessior^®^, a systemic fungicide based on boscalid and pyraclostrobin; Esquive^®^, a *Trichoderma atroviride*-based BCA; and Bentogran ^®^, which is composed of sodium bentonite, an inert compound.

## 2. Materials and Methods

### 2.1. The Vineyards

Treatments took place in the experimental vineyards of the Instituto Superior De Agronomia (Almotivo vineyard; 38°42′32.7′′ N, 9°11′11.5′′ W) in Lisbon, Portugal, and of the University of Turin (DISAFA vineyard; 45°3′53′′ N, 7°35′32′′ E) in Grugliasco (TO), Italy. Meteorological data are available in the Supplementary Materials. 

The Almotivo vineyard has a density of 3333 plants/ha, the soil is classified as vertisol, it is managed under conventional agricultural practices and there is no irrigation system. The selected cultivars were Cabernet Sauvignon and Syrah, both grafted on 140 RU rootstock (*Vitis berlandieri* × *Vitis rupestris*), trained as Cordon Royat bilateral and spur-pruned. Grapevines were planted in 1998 and were 24 years old at the time of sampling. The vineyard has a history of esca, with leaf-symptomatic grapevines accounting for ≤1% of the total plants in all recorded years (from 2015 to 2022). An in-depth microbiological analysis of fungal endophytic communities in Cabernet Sauvignon can be found in [34]. Symptoms correlated with other GTDs were also detected [35].

The DISAFA vineyard has a density of 4400 plants/ha and is located at 293 m above sea level in a plain area. The soil is sandy, further characteristics are detailed by Catoni et al. (2012) [36]. The vineyard is managed under conventional agricultural practices, and the irrigation system, although present, as it is an experimental vineyard, was not used in 2022. Cabernet Sauvignon was grafted on 779P (*Vitis berlandieri* × *Vitis rupestris*), and Syrah was grafted on SO4 (*Vitis berlandieri* × *Vitis riparia*). Vines are vertically trained and Guyot pruned. Grapevines were planted in 2008 and were 14 years old at the time of sampling. The vineyard is known to be affected by GTDs; however, no microbiological examination has been carried out.

### 2.2. Experimental Setup and Sample Processing

#### 2.2.1. Experimental Setup

**Figure 1:**
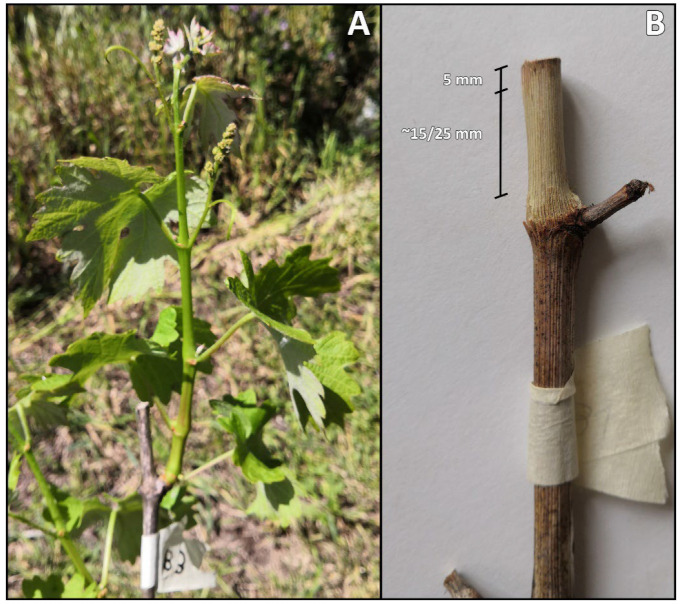
Grapevine shoot (cv. Syrah) at phenological stage 13—inflorescence clearly visible, 6 leaves separated—Almotivo vineyard (**A**). Treated grapevine cane deprived of the bark in the area of interest (**B**). The upper 5 mm of wood was discarded, while the following ~15 mm of wood was cut into small pieces and ground to dust.

Externally asymptomatic canes from vines that did not display GTD-associated symptoms in the 2021 growing season were selected and labelled during plant dormancy. Two hundred canes were pruned 2 to 3 cm above the fourth node (approx. 20 cm above the spur or above the fruiting cane) in January 2022 and monitored for the following three days. On the third day, canes with minimal or no bleeding were selected for treatment application. Each pruning wound protection product and a control (sterile distilled water, SDW) were applied to six canes, each corresponding to one biological replicate per cultivar per location (total *n* = 120). The PWPPs examined in this study are listed in Table 1. Using a micropipette, 25 μL of Cuprocol^®^, Esquive^®^, Tessior^®^ or SDW was deposited on the surface of pruning wounds and covered with Parafilm^®^, while sodium bentonite, an inert compound acting as a physical barrier, was applied with a brush, and no Parafilm^®^ was added. Cane collection took place upon reaching phenological stage 13—inflorescence clearly visible, 6 leaves separated (Figure 1A)—which occurred in April 2022 in the Almotivo vineyard (PT) and in May 2022 in the DISAFA vineyard (IT). A treatment-dependent delay in reaching the desired phenological stage was observed, in both vineyards, with Cuprocol-treated canes harvested 5 to 7 days after control canes.

While in the field, collected canes were kept on ice; then, they were frozen, freeze-dried and stored at −80 °C.

#### 2.2.2. Sample Processing

In a sterile environment, the upper 3 cm of cane bark was removed with the aid of a sterile scalpel, and the 5 mm of wood closest to the treated pruning wound was discarded (Figure 1B). Approximately 1.5 cm of wood was cut into small pieces with sterile pruning scissors and immediately ground to dust in sterile a mortar with the aid of liquid nitrogen.

### 2.3. DNA Extraction, Amplification, Library Preparation and Sequencing

For each sample, 150 mg of ground wood was used to extract genomic DNA, using the procedure described by Cenis (1992) [37], with minor modifications. Briefly, ground wood samples and extraction buffer were added to 1.5 mL Eppendorf tubes. The tubes were then vortexed for 1 minute and heated at 65 °C for 10 minutes. The DNA extraction protocol was then followed as per Cenis (1992) [37].

The library was prepared following a 2-step amplification protocol analogous to that described by Scibetta et al. (2018) [38]. The first amplification was conducted using primers ITS1-F-KYO2 (TCGTCGGCAGCGTCAGATGTGTATAAGAGACAG-TAGAGGAAGTAAAAGTCGTAA) and ITS86R (GTCTCGTGGGCTCGGAGATGTGTATAAGAGAC-TTCAAAGATTCGATGATTCAC) with Illumina^®^ (San Diego, CA, USA) overhangs [38]. Each reaction contained 10 uL of Platinum™ Hot Start PCR Master Mix (2×) (ThermoFisher Scientific, Waltham, MA, USA), 0.5 uL of primers (stock concentration of 10 mM), 1 uL of DNA template and 8 uL of PCR-grade water (Sigma-Aldrich, St. Louis, MO, USA). The PCR program for the first step was implemented as follows: 94 °C for 5′ followed by 39 cycles of 94 °C for 15′′, 55 °C for 15′′, 68 °C for 15′′ and, finally, a step at 68 °C for 5′. After the first PCR, the desired product was visualized on 1% agarose gel. A first PCR cleanup was carried out using AmpPure XP magnetic beads (Beckman Coulter, Brea, CA, USA) to remove the excess primer dimers. The second PCR step to index the products was carried out using 12.5 uL of Platinum™ Hot Start PCR Master Mix (2×) (ThermoFisher Scientific, Waltham, MA, USA), 2 uL of each Illumina^®^ barcode primer (stock at 5 mM), 5 uL of PCR1 product and 7.5 uL of PCR-grade water. The PCR cycle for the barcoding PCR was as follows: 94 °C for 5′ followed by 15 cycles of 94 °C for 15′′, 55 °C for 15′′, 68 °C for 15′′ and, finally, a step at 68 °C for 5′. After this second PCR amplification, cleanup was performed with magnetic beads using AmpPure XP, and the product was resuspended in 27 uL of PCR-grade water and verified on an 1% agarose gel. The libraries were then measured with Qubit (ThermoFisher Scientific, Waltham, MA, USA) using a high-sensitivity kit, then pooled in an equimolar amount. All the samples were sequenced on an Illumina^®^ MiSeq with 2 × 300 bp V3 Kit.

### 2.4. Bioinformatics

After sequencing, demultiplexing was performed with our Illumina^®^ MiSeq platform, and the raw data were analyzed using QIIME 2 v. 2022.2 [39] with the same pipeline described by Gobbi et al. (2019) [40]. The raw reads were imported and clipped to 12 bp on the 5′ end to remove the primers. Then, they were denoised using DADA2 [41]. Singletons were discarded. To minimize the effect of low-abundance amplicon sequence variants (ASVs) on the downstream statistics, all features that appeared less than 25 times in the dataset were filtered out. Taxonomic assignments were performed at 99% identity using QIIME feature classifier with BLAST in the UNITE [42] v9 database for ITS. After taxonomy assignment, the dominant features assigned to high taxonomical ranks such as order, class or family were further investigated using BLAST to refine the analyses in the NCBI database [43].

The raw data for this study are available in the European Nucleotide Archive (ENA accession number PRJEB60162).

### 2.5. Data Analysis

The resulting frequency table and its taxonomy were combined, converted to biom format in QIIME [39], then merged with a table of metadata into an S4 object and analyzed in R (version 3.6.3). The ‘phyloseq’ (version 1.30.0) [44] and ‘biomformat’ (version 1.14.0) [45] packages were used to create the primary data object. The ‘vegan’ (version 2.5.5) [46], ‘DESeq2’ (version 1.26.0) [47], ‘mvabund’ (version 4.1.3) [48], ‘metacoder’ (version 0.3.4) [49], ‘taxa’ (version 0.3.4) [50], ‘tidyverse’ (version 1.3.0) [51], ‘microbiome’ (version 1.8.0) [52], ggplot2, (version 3.3.2) [53] and ‘pairwiseAdonis’ (version 0.4.1) [54] packages were used for data manipulation, visualization and statistical analysis. The R code used for these analyses is publicly available at https://github.com/Marieag/LeaSyBiome/blob/main/LeaSyBiome_Study2.R (accessed on 15 February 2023).

The alpha diversity was measured using the Simpson (D) and inverse Simpson (1/D) diversity indices and tested with one-way ANOVA with post hoc Bonferroni correction to determine significant differences between cultivars and vineyards and among PWPP treatments.

We analyzed the β-dispersion to measure between-sample variances in abundance, computing the distances of group members from the group centroid. The resulting ordination was plotted using a Bray–Curtis distance matrix. To assess the overall intergroup variance (beta diversity), we also performed permutational multivariate analysis of variance (PERMANOVA) with 999 permutations, using the “vegan” package. Post hoc pairwise tests were performed to evaluate the differences between treatments using the “pairwiseAdonis” wrapper, applying FDR to correct for multiple comparisons. 

Additionally, we generated heat trees to visualize the effect size of the relative abundance of fungal taxa at different taxonomic levels using the ‘MetacodeR’ package, which calculates the log2 fold change (LfC) in genus and family abundance. A Wilcoxon rank sum test was applied to test differences between the same species in different tissue types or tissue groups, and the resulting *p*-values were corrected for multiple comparisons using FDR, as implemented in MetacodeR. We focused our analysis on taxa present at RA > 0.1%, and the *p*-value threshold was set to 0.05. We also used the DeSeq2 package to assess ASV differences between control water and other PWPPs, and any significant results were added to the heat tree in the form of stars at the relevant node tips. The main distinction between these two approaches is the method used to determine the middle data value: DeSeq2 uses overall counts (averages), which tends to favor less abundant ASVs, while MetacodeR uses between-sample counts (medians), which gives more weight to the presence of a given ASV in multiple samples. This difference in statistical approach highlights the benefits of applying both methods for a more comprehensive differential analysis.

## 3. Result

### 3.1. Sequencing Dataset Description

A total of 123 samples were sequenced, including positive and negative controls. The sum of forward and reverse reads amounts to 26,626,508, with an average of 216,476 reads/sample. After denoising, 7,094,778 high-quality ASVs were present in our dataset, with an average of 57,681 ASVs/sample representative of 938 dereplicated features. After discarding the controls and two samples, due to insufficient sequencing coverage, the remaining 118 samples were further analyzed.

### 3.2. The Wood Mycobiome

In the total dataset, 11 taxa are found at a relative abundance (RA) greater than 1%, 33 taxa at 1 < RA < 0.1% and 132 taxa at RA < 0.1% (total 176 taxa). The 44 most abundant taxa (i.e., RA > 0.1%) account for 97% of the dataset RA, and 30 out of 44 taxa were identified at the genus level (Table 2). Ascomycetes dominate the RA of the dataset (91.1%), followed by Basidiomycetes (8.4%) and others (0.5%). The most abundant Ascomycetes families are *Pleosporaceae* (34.2%), *Davidiellaceae* (16.9%) and *Aureobasidiaceae* (13.4%), while Basidiomycetes include families *Bulleribasidiaceae* (2.0%), *Filobasidiaceae* (1.4%) and *Sporidiobolaceae* (0.6%). To the best of our knowledge, among the taxa listed in Table 2, *Dioszegia, Buckleyzyma*, *Symmetrospora*, *Akenomyces, Papiliotrema* and *Kondoa* were detected for the first time in grapevine wood.

When comparing vineyards, among the top 44 taxa, 32 are shared between the Almotivo and DISAFA vineyards, 8 are unique to the former (e.g., *Stemphylium*, *Merismodes* and *Eutypella*) and 4 are unique to the latter (e.g., *Sarocladium* and *Neocucurbitaria*) (Table 2).

When comparing cultivars, among the 44 most abundant taxa, 40 are found in both Cabernet Sauvignon and Syrah; *Neocucurbitaria* and *Eutypella* are unique to the former, while *Akenomyces* and *Cystofilobasidium* are unique to the latter (Table 2).

Four GTD-associated taxa are detected among the RA > 0.1% taxa, i.e., *Cytospora*, *Phaeomoniella*, *Eutypella* and *Fusarium* [55,56]. Other GTD-associated taxa are also detected, albeit at lower relative abundances (RA < 0.1%), such as *Diplodia*, *Neofusicoccum* and *Diaporthe*.

### 3.3. Alpha Diversity

**Figure 2:**
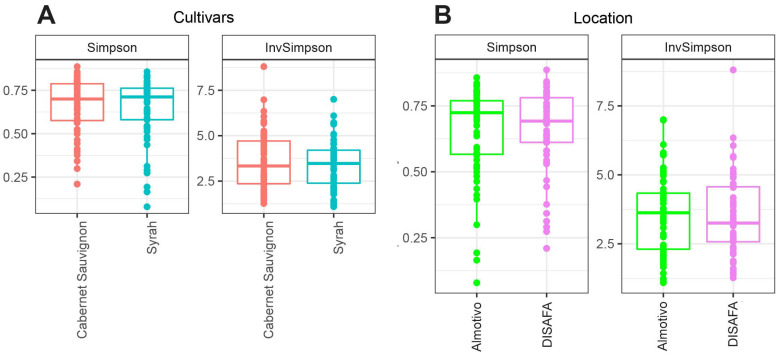
Box plots of alpha diversity indices (Simpson and InvSimpson) of the fungal communities in canes when analyzed by cultivar (Cabernet Sauvignon, Syrah; (**A**)) or vineyard (Almotivo, DISAFA; (**B**)).

The Simpson (D) and inverse Simpson (1/D) indices were used to assess the alpha diversity of the wood mycobiome. When comparing cultivars, according to one-way ANOVA, there are no significant differences between Cabernet Sauvignon and Syrah for both indices (*p* > 0.05; Figure 2A). Similarly, when comparing location, there are no significant differences between the Almotivo and DIFASA vineyards (*p* > 0.05; Figure 2B).

**Figure 3:**
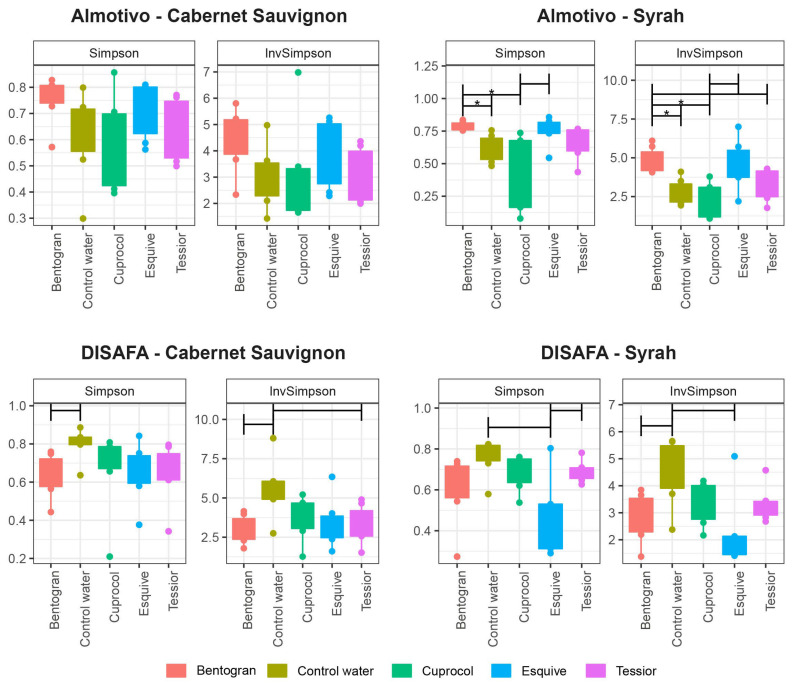
Box plots of alpha diversity indices (Simpson and InvSimpson) showing the richness of the fungal communities in canes treated with different pruning wound protection products (Bentogran, control water, Cuprocol, Esquive and Tessior) in Cabernet Sauvignon and Syrah from the Almotivo and DISAFA vineyards. The horizontal brackets marked with an asterisk (*) indicate statistical differences, while brackets without asterisks indicate trends (0.15 < *p* < 0.05).

When comparing the wood mycobiome of canes treated with different pruning wound protection products, the ANOVA showed significant differences in Syrah in the Almotivo vineyard (*p* < 0.05; Figure 3). Bentogran-treated canes are characterized by significantly greater D and 1/D values when compared to the control and Cuprocol-treated canes (both D and 1/D, *p* < 0.05). On the other hand, while non-significant, there are multiple trends (0.15 > *p* > 0.05) suggesting that the D and 1/D indices tend to be greater in control canes when compared to Bentogran (Cabernet Sauvignon, DISAFA vineyard) and Esquive treatments (Syrah, DISAFA vineyard) (Figure 3).

### 3.4. Beta Diversity

**Figure 4:**
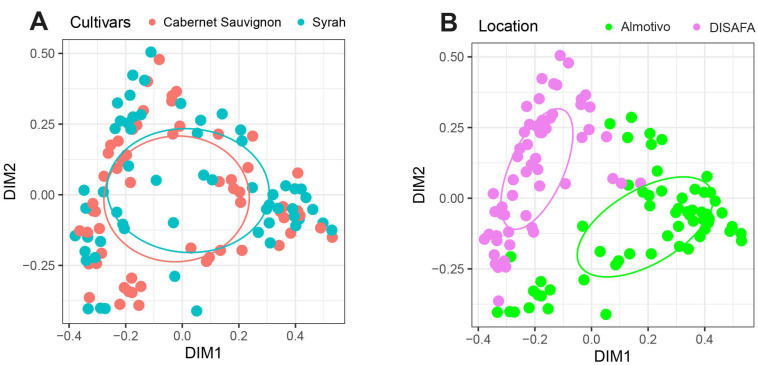
PCoA plots of beta dispersion based on Bray–Curtis dissimilarity of the fungal communities in canes when analyzed by cultivar (Cabernet Sauvignon, Syrah; (**A**)) or vineyard (Almotivo, DISAFA; (**B**)).

**Figure 5:**
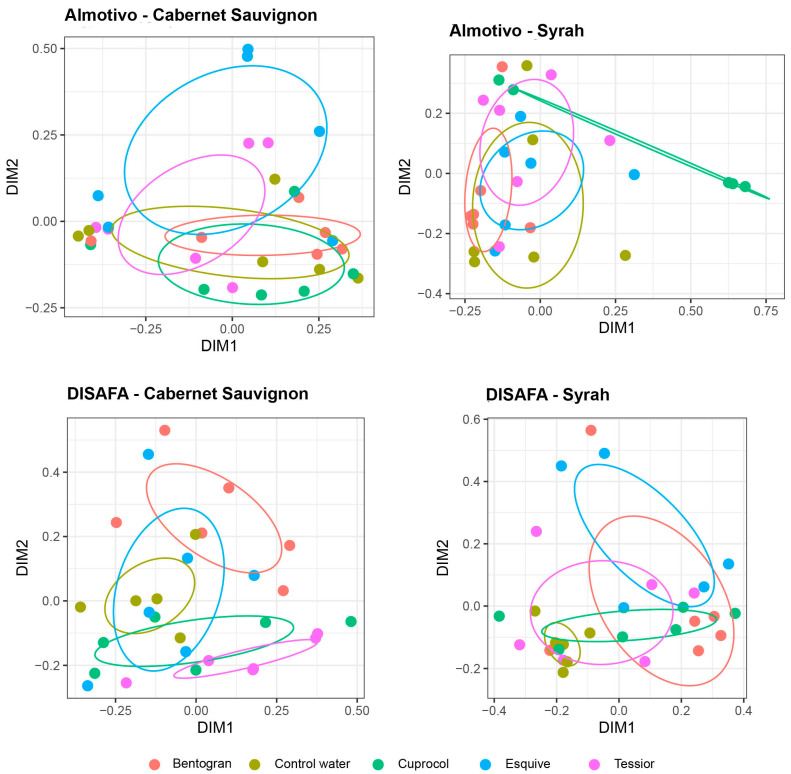
PCoA plots of beta dispersion based on the Bray–Curtis dissimilarity of the fungal communities in canes treated with different pruning wound protection products (Bentogran, Control water, Cuprocol, Esquive and Tessior) in Cabernet Sauvignon and Syrah from the Almotivo and DISAFA vineyards. Ellipses illustrate the multivariate normal distribution of samples within the same pruning wound protection product treatment.

The Bray–Curtis dissimilarity represented in PCoA plots of the beta dispersion was used to evaluate the beta diversity of the wood mycobiome (Figure 4 and Figure 5). When comparing cultivars, there are no significant differences between Cabernet Sauvignon and Syrah (*p* > 0.05; Figure 4A). On the contrary, when comparing location, according to PERMANOVA, there are highly significant differences between the Almotivo and DISAFA vineyards (*p* = 0.001; Figure 4B).

The PERMANOVA revealed significant treatment-dependent differences in the beta diversity of the wood mycobiome, albeit exclusively in samples from the DISAFA vineyard. In this vineyard, in Cabernet Sauvignon, the ‘pairwiseAdonis’ function revealed that the differences concern the Bentogran treatment, which clusters separately from Tessior (*p* = 0.01), control (*p* < 0.05) and Cuprocol treatments (*p* < 0.05), with Tessior also clustering separately from the control (*p* = 0.01) and Esquive treatments (*p* < 0.05) (Figure 5). Concerning Syrah, as visible from the clustering pattern, the Esquive treatment differs significantly from the control canes (*p* < 0.05).

In the Almotivo vineyard, treatments did not affect the beta diversity of the wood mycobiome in a significant manner. However, trends are observed in Syrah, where Esquive and Cuprocol treatments tend to cluster separately from Bentogran-treated canes (*p* = 0.095) (Figure 5).

### 3.5. Taxa over/under-Representation

**Figure 6:**
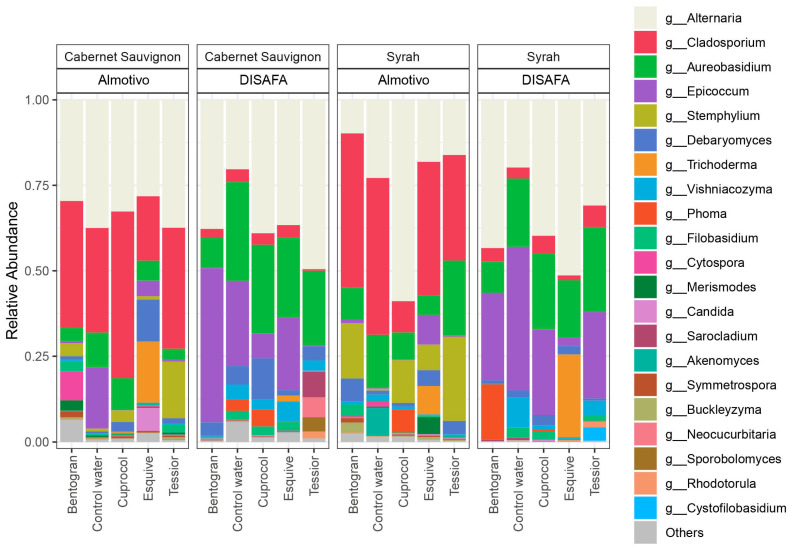
Bar plots of the relative abundance of the 21 most abundant taxa identified at the genus (g_) level found in canes treated with different pruning wound protection products (bentonite, Control water, Cuprocol, Esquive and Tessior) in Cabernet Sauvignon and Syrah from the Almotivo and DISAFA vineyards. ‘Others’ are taxa not included among the 21 most abundant.

On top of the information on the presence/absence of certain taxa (Table 2), the bar plot in Figure 6 provides an overview of key differences in taxa abundance depending on vineyard, cultivar and PWPP treatment. For example, *Cladosporium* is over-represented in the Almotivo vineyard (9-fold difference), while *Epicoccum* and *Vishniacozyma* are more abundant in the DISAFA vineyard (7-fold and 5-fold difference, respectively). Differences between cultivars are less pronounced, with *Stemphylium* and *Phoma* being more abundant in Syrah (2.5-fold difference) and *Candida* and *Cytospora* more abundant in Cabernet Sauvignon (4-fold difference).

**Figure 7:**
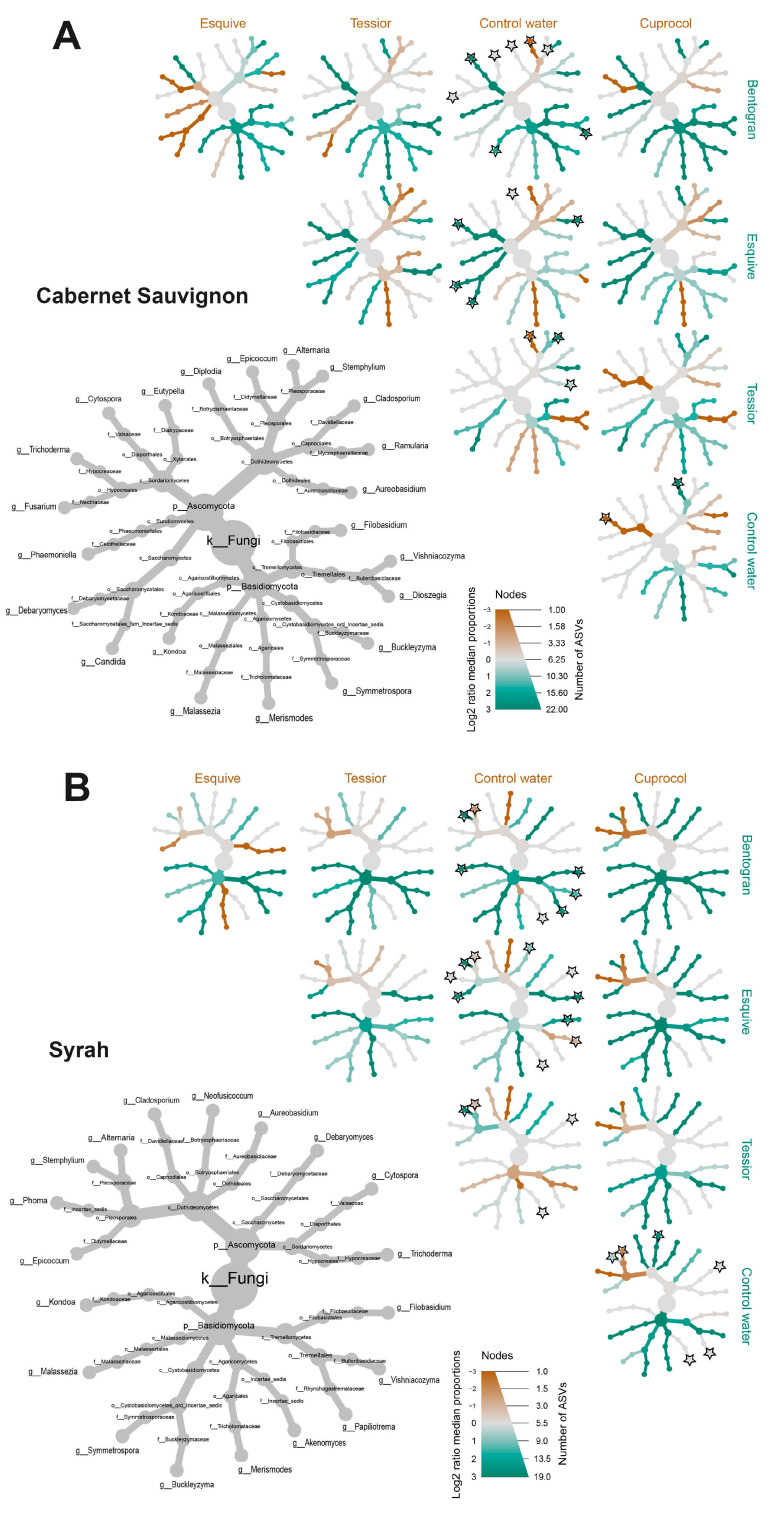
Differential heat tree matrices depicting changes in taxon abundance between different pruning wound protection product treatments (Bentogran, control water, Cuprocol, Esquive and Tessior) in Cabernet Sauvignon (**A**) and Syrah (**B**) from the Almotivo vineyard, represented in the dataset at a relative abundance (RA) > 0.1%. The smaller cladograms show pairwise comparisons between each treatment, with the color illustrating the log2 fold change: a green node indicates a lower abundance of the taxon in the treatment group on the abscissa than in the treatment group on the ordinate. A brown node indicates the opposite. A black star on a node represents statistically significant differences between treatments according to DeSeq2 (*p* < 0.05).

**Figure 8:**
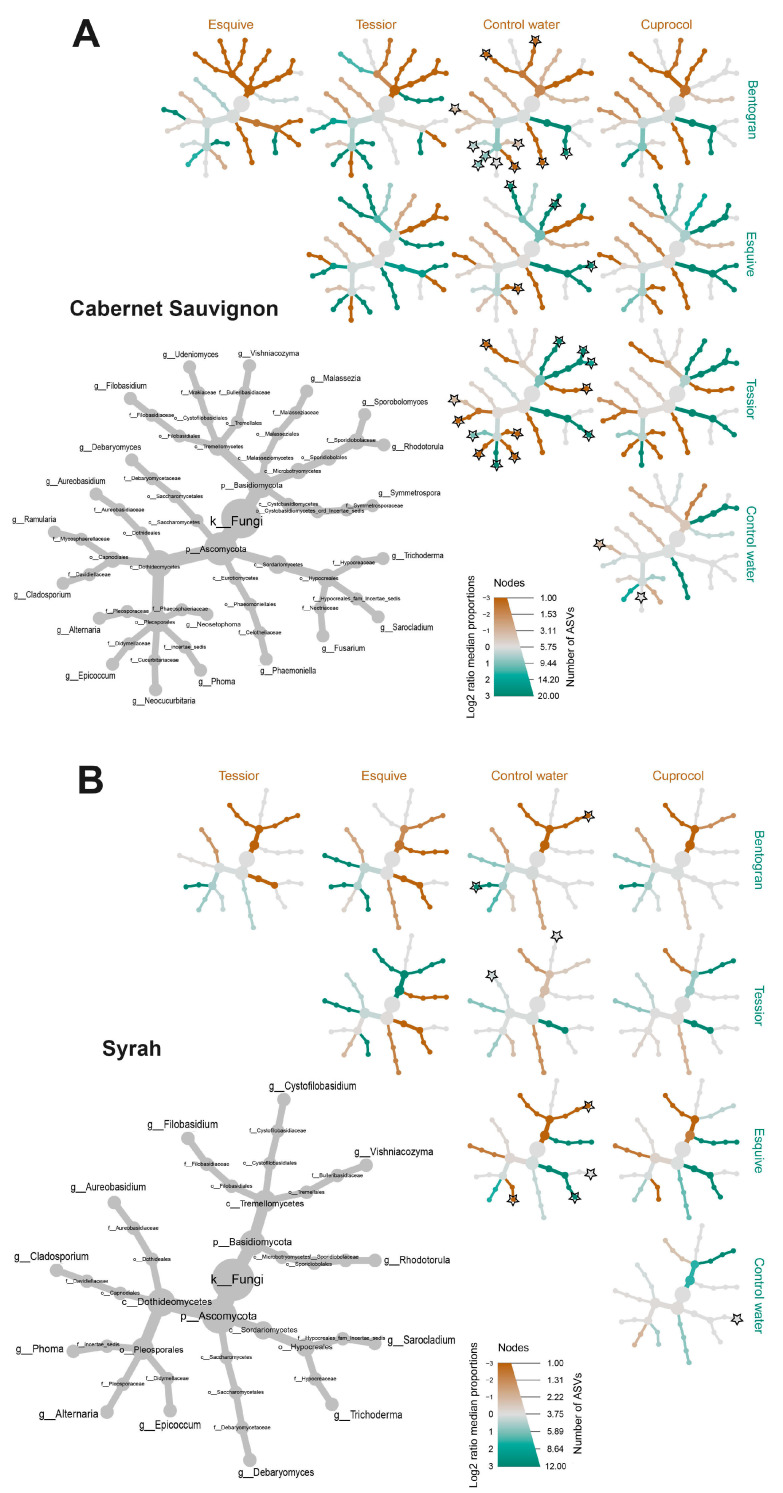
Differential heat tree matrices depicting changes in taxon abundance between different pruning wound protection product treatments (Bentogran, control water, Cuprocol, Esquive and Tessior) in Cabernet Sauvignon (**A**) and Syrah (**B**) from the DISAFA vineyard, represented in the dataset at a relative abundance (RA) > 0.1%. The smaller cladograms show pairwise comparisons between each treatment, with the color illustrating the log2 fold change: a green node indicates a lower abundance of the taxon in the treatment group on the abscissa than in the treatment group on the ordinate. A brown node indicates the opposite. A black star on a node represents statistically significant differences between treatments according to DeSeq2 (*p* < 0.05).

For an in-depth analysis of treatment-dependent alterations in taxa abundance, when compared to control canes, we employed MetacodeR (M; colored nodes in Figure 7 and Figure 8) and DeSeq2 (D; stars on nodes in Figure 7 and Figure 8) tests, focusing on taxa present at RA > 0.1% of the total dataset (Table 2). While examining the MetacodeR results, we focused on the taxa with the highest LfC (i.e., dark green and dark brown nodes).

PWPP treatments led to a greater amount of differently abundant taxa in the Almotivo vineyard (M = 41, D = 48) when compared to the DISAFA vineyard (M = 37, D = 36) and in Cabernet Sauvignon (M = 47, D = 47), when compared to Syrah (M = 31, D = 37). When focusing on specific PWPPs, to account for the artificial addition of *Trichoderma* via Esquive, we did not count this taxon among the over- or under-represented hits. The results show that Cuprocol application led to the fewest differently abundant taxa (M = 15, D = 12), followed by Esquive (M = 21, D = 19), Tessior (M = 19, D = 23) and bentonite (M = 19, D = 26) (Figure 7 and Figure 8).

Further examining the significant differences revealed by DeSeq2, taxa *Epicoccum*, *Alternaria*, *Stemphylium*, *Vishniacozyma* and *Phoma* are those most frequently affected by PWPP treatments, while *Debaryomyces* and *Cladosporium* are among those affected the least (Figure 7 and Figure 8).

## 4. Discussion

### 4.1. Mycobiome Composition

In recent years, grapevine wood mycobiome profiling has become of increasing interest due to its involvement in GTD [34,57,58] and its role in multiple biological processes that contribute to plant health [27,29]. NGS-based studies have revealed an unexpectedly wide fungal diversity—often greater than when estimated using culture-dependent approaches—and introduced new taxa not previously associated with grapevine wood, opening the debate on the function of rare taxa, while accurately estimating the relative abundances via amplicons [32,59,60].

Limited information is available on the mycobiome profile of one-year-old canes using culture-independent approaches. In a previous study conducted in the Almotivo vineyard, Del Frari et al. (2019a) revealed 54 taxa (total canes sampled n = 15) [34], while other researchers detected 138 AVS in France (n = 114) [61], 198 AVS in the United States (n = 60) [62] and 229 OTUs in Spain (n = 180) [63]. In the present study, we detected 176 taxa (n = 118), which is consistent with previous research. Nearly all studies that examined fungal communities of canes reported only a few taxa as dominant, the most frequent being *Alternaria*, *Cladosporium*, *Aureobasidium* and *Epicoccum* [34,61,63,64,65,66]. These ubiquitous, fast-growing Ascomycetes were also the most abundant species in the present study; they are considered early colonizers [65], and some of them have shown potential in the biological control of GTD-associated fungi [16,19,20]. Among other abundant taxa (RA > 1%), the ecological role of several genera, such as *Stemphylium*, *Vishniacozyma* and *Debaryomyces*, as well as their respective involvement in plant health processes and/or potential as biological control agents, remains unknown.

Mycobiome profiling shows that *Phaeomoniella chlamydospora* (identified as *Phaeomoniella* sp. in this study), a GTD pathogen that heavily affects the perennial wood of Cabernet Sauvignon in the Almotivo vineyard [34], and other GTD-associated taxa are detected at low RA values in canes, confirming a slow wood colonization process. This may be due to intrinsic temporal dynamics of fungal succession during wood colonization that occur with wood aging, as suggested by Kraus et al. (2019) [65].

Among non-rare taxa, we report six fungal genera as wood endophytes (*sensu* Hardoim [67]) for the first time: *Papiliotrema* and *Buckleyzyma*, which was previously detected in grape berries [68]; *Dioszegia, Symmetrospora* and *Kondoa* found in grapevine leaves [32,69,70]; and *Akenomyces,* a cryptic genus not yet reported in/on any grapevine organ. As mentioned in a previous study [34], we expect the list of first reports of grapevine wood endophytes to continue to grow with future NGS-based studies.

The geographical location of vineyards is known to be a strong predictor of fungal diversity in multiple grapevine organs [71,72,73,74], even if it remains unclear to what extent other parameters, such as the application of plant protection products, grapevine age and cultivar, contribute to observed differences. Despite this limitation, Bekris et al. (2021) reported significant differences in the wood mycobiome profile in Greek viticultural areas [73], and Martinez-Diz et al. (2020) found highly significant differences in alpha and beta diversity when comparing one-year-old canes in Spanish vineyards [63]. In this study, the significant differences in mycobiome beta diversity (Figure 2) revealed when comparing Almotivo and DISAFA vineyards confirm a major effect of sampling location. However, in addition to different geographical location, climate and *terroir*, numerous other factors may have contributed to the observed variability, such as rootstocks and training systems.

Grapevine genotype has been reported to influence the mycobiome profile in the rhizosphere of mature but not young vineyards [74], and it is variably (strong, weak or non-significant) correlated with fungal communities at phyllosphere level [71,72,75]. Differences in the microbial composition of rootstocks have been reported in multiple studies [76,77,78], although they seem to mostly concern individual taxa abundance rather than community indices. In the perennial wood of adult vines, Travadon et al. (2016) reported a large number of taxa unique to specific cultivars [79], and Bekris et al. (2021) revealed cultivar/viticultural zone-dependent differences [73]. In young vines, Lade et al. (2022) reported weak differences among cultivars at the nursery level [77], while in canes, Pancher et al. (2012) did not observe differences in fungal communities when comparing cultivars [66]. In the present study, which involved one-year-old canes, the genotype effect seems to be minor, as no significant differences were observed for the examined diversity indices when comparing Cabernet Sauvignon and Syrah. While some taxa seem to preferentially colonize specific cultivars (Table 2; Figure 6), it is unclear whether it is indeed a genotype-driven effect or only a result of the fortuitous presence of opportunistic fungi. Nevertheless, considering the abovementioned studies, grapevine genotype may play relevant roles in shaping the endophytic mycobiome of perennial wood in adult plants rather than annual wood or in young vines, and more research is needed to explore this topic.

### 4.2. Effects of Pruning Wound Protection Products on Mycobiome Profile

This study shows that some community indices used to evaluate alpha and beta diversity of the wood mycobiome vary significantly in response to PWPP treatment, although statistical significance is often strongly dependent on the cultivar and/or vineyard under examination. For example, alpha diversity indices differed significantly only in Syrah-Almotivo, while beta diversity was significantly different in both cultivars only in DISAFA (Figure 3 and Figure 5). When looking at individual taxa, all PWPP treatments led to significant alterations in the abundance of multiple fungi when compared to control canes. Among them, we found beneficial fungi (i.e., *Epicoccum,* [20]) and numerous others whose role in grapevine wood remains unknown (Figure 7 and Figure 8). These results highlight that mycobiome alterations do occur in response to PWPP treatment, both in terms of community indices and individual taxa and that they are strongly influenced by intrinsic mycobiome characteristics and other factors such as cultivar and geographical location.

#### 4.2.1. Cuprocol^®^

Despite its popularity among viticulturists, there is limited evidence suggesting that copper oxychloride and other cupric fungicides are effective as PWPPs [9,16,80,81]. Evidence shows that copper-treated vines may develop longer necrotic lesions and/or darker wood streaking when infected with some GTD-associated fungi [30,80,81], and symptoms may be present even in copper-treated but non-inoculated vines [30]. In the present study, in Cuprocol-treated canes, we noticed a delay in reaching our target phenological stage, as well as brown wood streaking-like symptoms extending up to 10 mm from the treatment area (data not shown), which suggests phytotoxic effects. We detected significant alterations in both alpha and beta diversity when comparing Cuprocol-treated canes with other PWPPs (Figure 3 and Figure 5). Individual taxa seem to be less affected by this treatment (Figure 7 and Figure 8), although a beneficial genus such as *Epicoccum* is significantly under-represented in Cabernet Sauvignon.

#### 4.2.2. Tessior^®^

The efficacy of boscalid and pyraclostrobin in fighting GTD pathogens has been positively evaluated in several studies [9,10,15]. In this study, a significant alteration in beta diversity and in individual taxa abundance demonstrates that this PWPP affects the wood mycobiome, as especially evident in *Epicoccum*, a genus significantly under-represented in Tessior-treated canes in Cabernet Sauvignon.

#### 4.2.3. Esquive^®^

Multiple *Trichoderma atroviride* strains have been validated as effective biological control agents that prevent GTD pathogens from infecting pruning wounds [9,10,26]. The results of mycobiome profiling show that the use of this BCA may affect community indices of alpha diversity (trends) and beta diversity (significantly), albeit exclusively in Syrah (Figure 3 and Figure 5). Unsurprisingly, several fungal genera are under- or over-represented in Esquive-treated canes; however, *Epicoccum* and other beneficial fungi seem to be less negatively affected when compared with Cuprocol and Tessior-treated canes.

#### 4.2.4. Bentogran^®^

Sodium bentonite has shown promising results in pruning wound protection in recent field trials [82], and further research is currently underway. If its efficacy is confirmed, sodium bentonite may represent a valid environmentally friendly alternative to other PWPPs. In this study, bentonite significantly increased the alpha diversity indices in the Almotivo vineyard, which is generally regarded as a positive factor for community stability [83]. However, this was not replicated in the DISAFA vineyard, where, on the contrary, the trend seems opposite (Figure 3). This treatment led to alterations in beta diversity (Figure 5) and in the abundance of numerous taxa (Figure 7 and Figure 8). Hypothetically, the hygroscopic characteristic of this clay, i.e., the capacity to absorb and retaining moisture, may have created a favorable environment for the proliferation of certain fungi. Whether this mycobiome alteration indirectly leads to increased resistance to fungal colonizers (including GTD fungi) remains to be evaluated.

Covering treated canes with a physical barrier allowed us to focus on the effect of PWPPs on the fungal communities already present in the wood. In future studies, the role that PWPPs play in the wood colonization success of fungi that naturally reach pruning wounds under vineyard conditions, with or without the simultaneous inoculation of GTD pathogens, shall be evaluated. As demonstrated by Del Frari et al. (2019b), some fungicides alter the wood colonization success of common endophytes, e.g., *Alternaria alternata, Aureobasidium pullulans* and *Epicoccum nigrum* [30], which may compromise microbial dynamics such as those suggested by Kraus et al. (2019) [65]. In addition, future studies will evaluate whether PWPP-induced alteration in the mycobiome profile are long-lasting and dependent on other factors such as climatic conditions and vineyard management, among others. This is especially relevant for biological control agents, such as those based on *Trichoderma* spp., the efficacy of which is known to be influenced by multiple factors and may vary from year to year [5,21,23,26].

In this paper, we presented evidence suggesting that PWPP-induced alterations in the mycobiome profile and/or taxa abundance are strictly correlated with the mycobiome composition itself, which may vary depending on the cultivar and the geographic location of vineyards. However, yearly variations in the grapevine-associated mycobiome have been reported in recent studies [58,62,84]. While it remains unclear to what extent yearly variations influence the microbiological profile of annual wood, we believe that this additional factor should be considered to achieve a more comprehensive understanding of the issue.

## 5. Conclusions

Pruning wound protection products are considered the best currently available means of reducing GTD-associated pathogen infections under vineyard conditions. Nevertheless, some PWPPs present certain challenges that prevent their widespread application. Delivery means should be optimized, economic advantages should be clearly evaluated and environmental consequences should be better understood. Concerning the latter, our results show that some PWPPs can significantly affect fungal communities residing in grapevine wood. It remains unclear whether such alterations lead to substantial negative consequences for the well-being of grapevines, and it will be a priority to evaluate these aspects in future studies. Based on the results of this study, we believe it is too early to recommend any specific PWPP, and more research is necessary to deepen our understanding of the multiple factors involved in grapevine–mycobiome–PWPP interactions.

## Figures and Tables

**Table 1:** Pruning wound protection products applied to three-day-old pruning wounds.

Trade Name	Manufacturer	Active Ingredient/BCA	Tested Concentration
Cuprocol^®^	Syngenta (Basel, Switzerland)	Copper oxychloride	36.5 g L^−1^
Esquive^®^	Agrauxine (Marcq-en-Barœul, France)	*Trichoderma atroviride* I-1237 (1 × 10^8^ CFU g^−1^)	100.0 g L^−1^
Tessior^®^	BASF (Ludwigshafen, Germany)	Boscalid 10 g L^−1^ + Pyraclostrobin 5 g L^−1^	10.0 g L^−1^ boscalid + 5.0 g L^−1^ pyraclostrobin
Bentogran^®^	AEB Bioq. Port. (Singapore)	Sodium bentonite	80% (*v/v*) sodium bentonite in water

**Table 2:** Taxonomic classification of the 30 most abundant taxa identified at the genus level and found at a relative abundance (RA) equal to or greater than 0.1% of the total dataset. Presence (+) or absence (−) of individual taxa is displayed when comparing vineyards (Almotivo, DISAFA) and cultivars (Cabernet Sauvignon, Syrah) and when examining GTD-associated fungi (GTD).

Phylum	Family	Genus	RA (%)	Vineyard		Cultivar		GTD
				Almotivo	DISAFA	Cabernet Sauvignon	Syrah	
Ascomycetes	*Pleosporaceae*	*Alternaria*	30.0	+	+	+	+	−
		*Stemphylium*	4.0	+	−	+	+	−
	*Davidiellaceae*	*Cladosporium*	17.0	+	+	+	+	−
	*Aureobasidiaceae*	*Aureobasidium*	13.4	+	+	+	+	−
	*Didymellaceae*	*Epicoccum*	11.6	+	+	+	+	−
		*Phoma*	1.4	+	+	+	+	−
	*Debaryomycetaceae*	*Debaryomyces*	3.3	+	+	+	+	−
	*Hypocreaceae*	*Trichoderma*	2.3	+	+	+	+	−
	*Saccharomycetaceae*	*Candida*	0.7	+	+	+	+	−
	*Valsaceae*	*Cytospora*	0.5	+	-	+	+	+
	*Hypocreales*	*Sarocladium*	0.5	−	+	+	+	−
	*Cucurbitariaceae*	*Neocucurbitaria*	0.3	−	+	+	-	−
	*Phaeosphaeriaceae*	*Neosetophoma*	0.2	+	+	+	+	−
	*Phaeomoniellaceae*	*Phaemoniella*	0.2	+	+	+	+	+
	*Mycosphaerellaceae*	*Ramularia*	0.1	+	+	+	+	−
	*Diatrypaceae*	*Eutypella*	0.1	+	−	+	−	+
	*Nectriaceae*	*Fusarium*	0.1	+	+	+	+	+ *
Basidiomycetes	*Bulleribasidiaceae*	*Vishniacozyma*	1.8	+	+	+	+	−
		*Dioszegia* †	0.1	+	+	+	+	−
	*Filobasidiaceae*	*Filobasidium*	1.3	+	+	+	+	−
	*Tricholomataceae*	*Merismodes*	0.5	+	-	+	+	−
	*Buckleyzymaceae*	*Buckleyzyma* †	0.4	+	+	+	+	−
	*Malasseziaceae*	*Malassezia*	0.4	+	+	+	+	−
	*Symmetrosporaceae*	*Symmetrospora* †	0.4	+	+	+	+	−
	*Sporidiobolaceae*	*Sporobolomyces*	0.3	+	+	+	+	−
		*Rhodotorula*	0.2	+	+	+	+	−
	n/a	*Akenomyces* †	0.3	+	+	−	+	−
	*Cystofilobasidiaceae*	*Cystofilobasidium*	0.3	−	+	−	+	−
	*Rhynchogastremataceae*	*Papiliotrema* †	0.2	+	+	+	+	−
	*Kondoaceae*	*Kondoa* †	0.2	+	−	+	+	−

† First report in grapevine wood; * the genus *Fusarium* contains both pathogenic and non-pathogenic species.

## Data Availability

The raw data for this study are available in the European Nucleotide Archive (ENA accession number PRJEB60162).

## Supplementary Materials

The following supporting information can be downloaded at: https://www.mdpi.com/article/10.3390/jof9040488/s1, Table S1. Meteorological data of the DISAFA and Almotivo vineyards during the first five months of 2022.
